# Tumor Suppressors and Endodermal Differentiation of P19 Embryonic Stem Cells

**DOI:** 10.4172/2168-9296.1000e138

**Published:** 2015-12-30

**Authors:** Jyotshna Kanungo

**Affiliations:** Division of Neurotoxicology, National Center for Toxicological Research, US Food and Drug Administration, Jefferson, AR 72079, USA

**Keywords:** Retinoic acid, P19 ES cell, Tumor suppressors, Endodermal differentiation

The P19 embryonic stem (ES) cells are derivatives of the inner cell mass of a mouse blastoderm, are multipotent and can give rise to all three germ layers [1]. They are anchorage-independent, display no contact inhibition, and are tumorigenic [2]. The P19 ES cell line was originally derived from a teratocarcinoma in C3H/HE mice, produced by grafting an embryo at 7 days of gestation to the testes of an adult male mouse [3, 4]. Depending on the nature of inducers, P19 ES cells can be driven to differentiate into derivatives of all three germ layers, an advantage that has been extensively exploited to study early developmental events. Dimethyl sulfoxide (DMSO) treatment of P19 ES cell aggregates (embryoid bodies) results in differentiation into cardiac- and skeletal muscle-like cells [1], whereas retinoic acid (RA) induces differentiation into neurons, glia, and fibroblast-like cells [5]. On the other hand, monolayers of P19 ES cells, when treated with RA, differentiate into cells with endodermal and mesodermal phenotypes [6]. The type of differentiation of P19 ES cell aggregates also depends on the RA concentration; with low concentration (10 nM) of RA, these cells differentiate into primitive endoderm-like cells and with high concentrations (1 µM) of RA, differentiation is shifted towards neurons and glia [3, 7, 8].

Although extensive studies on RA-induced neuronal differentiation of P19 ES cells exist [9–12], very few studies report specific gene/protein-induced endodermal differentiation. For example, endodermal differentiation of P19 ES cells requiring G-proteins, such as G_α13_ and G_α12_ [13–15], JLP (JNK-interacting leucine zipper protein), a scaffold protein [16], a LIM-protein, Ajuba [17] and a tumor suppressor, Menin [18], has been shown.

Tumor suppressors are characterized as proteins whose expression or activity needs to be attenuated for a cell to become cancerous [19]. In P19 ES cells, endodermal differentiation mediated by two tumor suppressors, Ku and Menin, has been reported [18, 20]. Ku is primarily involved in DNA repair and non-homologous recombination and is the heterodimeric regulatory component of the serine/threonine kinase, DNA-dependent protein kinase (DNA-PK) [21]. Ku consists of 80 (Ku80) and 70 kDa (Ku70) subunits [22]. Ku80 is also a somatostatin receptor that can regulate the activity of protein phosphatase 2A (PP2A) [23]. The fact that somatostatin is an inhibitor of cell proliferation and that PP2A is involved in cell cycle regulation [24] validated Ku80 as a suppressor of cell growth.

Ku reportedly inhibits rDNA transcription [25]. Retarded cell growth by Ku via repression of RNA polymerase I-mediated transcription has been demonstrated [26, 27]. Ku mediates the repression of mouse ribosomal gene transcription [28], and a member of the Ku protein family, non-histone protein 1 (NHP1) has been shown to be upregulated in differentiation of mouse myoblasts and human promyelocytes [29]. Furthermore, inhibition of the Ku heterodimer DNA binding activity, while the Ku protein level remained unaltered, was linked to granulocytic differentiation of human promyelocytic cell lines [30]. Report on the RNA polymerase I transcription-suppressive effects of Ku presented compelling evidence that Ku, directly or indirectly, could affect cell growth [31], and in turn may induce cell differentiation.

It has been reported that constitutively active G_α12_ and G_α13_ induced endodermal differentiation of P19 ES cells [13, 14] by modulating the MEKK4/JNK1 signaling pathway [15, 32]. Co-expression of an antisense Ku80 (AS-Ku80) reduced Ku80 expression in constitutively active G_α13_ (G_α13_Q226L)-expressing cells and inhibited endodermal differentiation. The level of Ku70 also decreased in these cells indicating that the loss of one of the Ku subunits results in the loss of the other subunit [20]. This interdependence of the two Ku subunits for their stabilization has been reported [33, 34]. Overexpression of either G_α13_ Q226L or Ku80 down-regulated RNA polymerase I-mediated transcriptional activity whereas co-expression of AS-Ku80 restored the activity to control levels [20], but abrogated G_α13_-mediated endodermal differentiation in P19 ES cells, indicating a critical role of Ku-80. However, Ku80 was not sufficient to induce endodermal differentiation in these cells [20] suggesting that Ku80 may be an indispensable protein downstream of G_α13_ Q226L signaling required for the endodermal differentiation of P19 ES cells [20].

Another tumor suppressor, Menin is a 61 kDa nuclear protein [35]. It is the product of the multiple endocrine neoplasia type I (*Men1*) gene, mutations of which, are known to cause the human autosomal dominant syndrome with development of tumors of the parathyroid, endocrine pancreas, and anterior pituitary [36]. A ubiquitously expressed protein, Menin bears no homology to functionally identified domains, but binds to JunD thus attenuating cell growth [37]. *Men1*-null mice are embryonically lethal suggesting the cause to be early developmental defects [38]. *Men1*-null embryonic fibroblasts enter senescence earlier than their wild-type counterparts and *Men1*-null ES cells can not form embryoid bodies suggesting an impaired differentiation capacity of these cells [38]. Menin’s role in duct cell differentiation in mouse submandibular gland [39], and in early differentiation of osteoblasts but inhibition of their later differentiation, has been reported [40, 41]. Menin influences *Hoxa9* gene expression and thereby regulates hematopoiesis and myeloid transformation [42, 43].

In P19 ES cells, RA modulated Menin expression, reduced cell growth and induced endodermal differentiation [18]. Although *Men1* over-expression suppressed P19 ES cell growth, the cells did not undergo endodermal differentiation in monolayer cultures, but did so upon cell aggregation. When aggregated in the presence of RA, these cells formed smaller embryoid bodies compared to the untreated ones and eventually underwent apoptosis [18]. Since endodermal differentiation occurred without RA in the P19 ES cell aggregates, the requirement of cell aggregation for Menin to induce endodermal differentiation in the absence of RA was hypothesized [18].

RA first binds to its nuclear receptor RARα (retinoic acid receptor alpha) and then triggers the transcription of other downstream RARs, especially the RA-receptor and tumor suppressor, RARβ2 [44]. In the absence of RARα, RA cannot execute its growth inhibitory effect [45]. Whether RA-induction of Menin expression in P19 ES cell aggregates, but not in cell monolayers, depended on RARα−mediated RARβ2 activation regulating *Men1* transcription is not clear. However, Menin upregulated the mRNA of the three *RAR*s (*RARα, RARβ* and *RARγ*) in the P19 ES cell aggregates (embryoid bodies), but not in monolayers [18]. These findings indicated that not only is *Men1* an RA-responsive gene, but it also, in turn, induces the expression of the *RAR*s. Induction of the expression of the *RAR*s by Menin may be linked to the endodermal differentiation of P19 ES cells [18]. It’s known that RA’s differentiation-inducing function is mediated by ligand-dependent activation of the specific RARs. Therefore, Menin could activate the RARs in an RA-independent manner and thus result in endodermal differentiation of the P19 ES cells. For example, over-expression of either *Ngn1* or *Sox6* or *Stra13* has been shown to be sufficient to induce neuronal differentiation of P19 ES cells in the absence of RA [46–48]. Also in the absence of DMSO, certain transcription factors that induce mesodermal differentiation upon their over-expression in P19 ES cells include MEF2C and Nkx2—5 [49], GATA-4 [50], MyoD [51] and β-catenin [52]. In the embryoid bodies, only 10–20% of *Men 1* over-expressing P19 ES cells at the core region underwent endodermal differentiation [18] indicating that Menin could regulate cellular differentiation that’s co-dependent on cell microenvironment, cell adhesion, and inter-cellular signaling, etc. Therefore, Menin’s interaction with other unidentified players in these biological processes (aggregation followed by endodermal differentiation) seems obvious. While Menin was sufficient to induce endodermal differentiation in aggregated P19 ES cells, the differentiation was inhibited by the pan-RAR antagonist Ro41-5253. Whether Menin regulates the RARs’ transcriptional activation potential remains to be examined and so is the mechanism of the regulation of other downstream targets that are critical for endodermal differentiation. In summary, the study presented evidence that Menin, a known tumor suppressor, is a key player in the RA signaling pathway and is critical for endodermal differentiation [18].

P19 ES cells continue to serve as an ideal model system to study how various gene products including tumor suppressors affect early embryonic development and identify the mechanism(s) that regulate it. Most importantly, when a gene deletion or over-expression causes embryonic lethality thus prohibiting further studies on early developmental events, P19 ES cells can be successfully utilized instead to recapitulate the early embryonic developmental processes. In addition, understanding the mechanism by which the tumor suppressors are regulated by the morphogen, RA, or the way they themselves regulate RA function by modulating the RARs, may prove useful in developing retinoid-based therapies for various diseases, especially cancer.
